# Reliable Surface Analysis Data of Nanomaterials in Support of Risk Assessment Based on Minimum Information Requirements

**DOI:** 10.3390/nano11030639

**Published:** 2021-03-05

**Authors:** Jörg Radnik, Reinhard Kersting, Birgit Hagenhoff, Francesca Bennet, Dmitri Ciornii, Penny Nymark, Roland Grafström, Vasile-Dan Hodoroaba

**Affiliations:** 1 Bundesanstalt für Materialforschung und-Prüfung (BAM), Division 6.1 Surface Analysis and Interfacial Chemistry, Unter den Eichen 87, 12205 Berlin, Germany; Francesca.Bennet@bam.de (F.B.); Dmitri.Ciornii@bam.de (D.C.); dan.hodoroaba@bam.de (V.-D.H.); 2 Tascon GmbH, Mendelstr. 17, 48149 Münster, Germany; reinhard.kersting@tascon-gmbh.de (R.K.); birgit.hagenhoff@tascon-gmbh.de (B.H.); 3 Department of Toxicology, Misvik Biology, Karjakatu 35, 20520 Turku, Finland; penny.nymark@ki.se (P.N.); Roland.grafstrom@ki.se (R.G.); 4 Institute of Environmental Medicine, Karolinska Institute, Nobels väg 13, 17177 Stockholm, Sweden

**Keywords:** surface analysis, electron microscopy, energy dispersive X-ray spectroscopy (EDS), X-ray photoelectron spectroscopy (XPS), secondary ion mass spectrometry (SIMS), standardization, data formats, reproducibility crisis, risk assessment

## Abstract

The minimum information requirements needed to guarantee high-quality surface analysis data of nanomaterials are described with the aim to provide reliable and traceable information about size, shape, elemental composition and surface chemistry for risk assessment approaches. The widespread surface analysis methods electron microscopy (SEM), energy dispersive X-ray spectroscopy (EDS), X-ray photoelectron spectroscopy (XPS) and secondary ion mass spectrometry (SIMS) were considered. The complete analysis sequence from sample preparation, over measurements, to data analysis and data format for reporting and archiving is outlined. All selected methods are used in surface analysis since many years so that many aspects of the analysis (including (meta)data formats) are already standardized. As a practical analysis use case, two coated TiO_2_ reference nanoparticulate samples, which are available on the Joint Research Centre (JRC) repository, were selected. The added value of the complementary analysis is highlighted based on the minimum information requirements, which are well-defined for the analysis methods selected. The present paper is supposed to serve primarily as a source of understanding of the high standardization level already available for the high-quality data in surface analysis of nanomaterials as reliable input for the nanosafety community.

## 1. Introduction

Nanomaterials are widely used in various industrial processes and consumer products, e.g., catalysis, refinery, electronics, food, packaging, cosmetics, pharmaceutical and medical devices. A further expansion is expected in the forthcoming years [1,2,3,4]. Due to this wide distribution, the impact of nanomaterials on health and environment becomes more and more relevant [5,6]. The European legislation has responded to this development and defined the term “nanoform” in the Annexes to the REACH (registration, evaluation, authorization of chemicals) regulation [7]. Now, specific information of the nanomaterials is required from the companies when registering the appropriate materials in a dossier. The progress reached in the last decade has led to the establishment of a new branch in toxicology, the so-called nanotoxicology, which is specialized on questions like in what doses and how nanomaterials interact with biomolecules and the environment, how they are transformed by the biological systems and over what period of time. According to Bohnsack et al., nanotoxicology has to begin with comprehensive physical chemistry behind the nanomaterial under study [8]. For example, nanoparticles can agglomerate or aggregate, which may influence deposition and transport of those into/to different body organs; clearance from the body may be also altered if these are agglomerated/aggregated, etc. [9]. This process of agglomeration/aggregation is determined by the nanoparticle size and concentration: “the smaller the particles and the higher the concentration, the greater the aggregation/agglomeration rate” [10]. Despite great efforts in the nano-risk research area and large number of publications on nanotoxicology during the last decade, there are many challenges that nanotoxicology is facing, e.g.:

- The influence the different environments such as air, soils or sediments, freshwater or seawater on the toxicity of the nanomaterials,
- Categorization and prioritization of different types of nanomaterials for the ecotoxicological risk assessments [11] (grouping and read across),
- The increased complexity of nanomaterials, e.g., core–shell particles [12].

In the context of REACH, eleven physicochemical properties were considered as relevant, of which the following six are essential for registration of nanoforms (priority properties): chemical composition, crystallinity, particle size, particle shape, chemical nature of the surface (“surface chemistry”) and specific surface area (SSA) [7]. This key role of these properties stresses the importance of reliable, reproducible and traceable data. This need is boosted by the consideration of ECHA (The European Chemicals Agency) to use them potentially for grouping and read across [13]. Obviously, all efforts in this field and more generally, in nanoinformatics for risk assessment, can only be successful, if the data, which are used are not only available and complete, but also of guaranteed high quality.

The quality of physicochemical data is discussed in the literature in terms of completeness, relevance (meaningfulness and usefulness), reliability (trustworthiness), accessibility/availability and reproducibility [14]. Data completeness in nanotoxicology refers to availability of a complete physicochemical characterization of the nanomaterial (including also raw, derived and meta-data) and an extensive description of the experiments and methods. In a regulatory context, for example, complete information is then considered if the information allows a regulatory decision. One way to evaluate data completeness is to employ a minimum information checklist (examples of such checklists were already reported in the literature) [15] and to estimate the compliance with such a checklist. One has to mention here that data completeness may be considered highly context-dependent.

Data relevance may be regarded as “fit-for-purpose”, which tells whether data serve to clarify particular question/purpose, not necessarily being useful for answering other questions. As such, only relevant data should be taken into account when assessing data completeness [15].

Reliability of the data is another criterion that means data trustworthiness or “credibility”. Mainly, data can be considered reliable if these are compliant with Good Laboratory Practice or other standardized protocols, like guidelines of the Organization for Economic Co-operation and Development (OECD) or rulings of the International Organization for Standardization (ISO) [16]. important aspect in assigning data as reliable is the evaluation of an intrinsic scientific quality of the data. This aspect is not always addressed in the literature; however, we consider that only an expert team with long-term experience can judge about scientific quality (reliability) of the experimental data. Best assumptions would be achieved if different expert-teams (from different accredited laboratories) evaluate scientific quality of the data.

For the data availability and reproducibility, the FAIR (findability, accessibility, interoperability and reusability) guiding principles for scientific data management give minimal requirements for finding, accessing, (re)using and citing scientific data [17]. In times of generating big quantities of data (“Big data”) such discussion is necessary, particularly, how to open data for other interested parties and avoid unnecessary time-wasting measurements.

On the other hand, the community has been discussing the “reproducibility crisis” for several years [18]. To overcome this crisis and to accelerate nanotechnology research, scientific publishers and more and more project funding agencies encourage authors to publish data in suitable repositories and to respect the FAIR principles [19,20,21]. It can be hoped that such sustained efforts coming from the whole scientific community contribute to solving the “reproducibility crisis” [22]. The consideration of such guidelines is not only necessary for scientific publications, but also even more for data used for risk assessment. Therefore, we demonstrate for the physicochemical properties particle size, particle shape, chemical composition and surface chemistry with representative examples of nanoparticles, how the data quality can be ensured such that risk assessment can be made in a reliable way.

**Figure 1:**
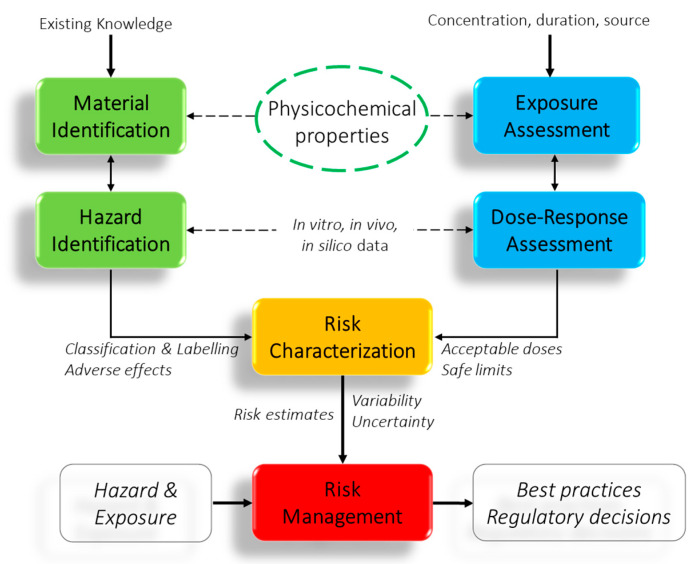
Physicochemical properties in the context of risk assessment (adapted from [25]).

As mentioned before, the first step in nanotoxicology is the complete characterization of the nanomaterial, since further toxicological interpretation is based on these data. If the extensive and robust characterization is lacking, the linking between nanomaterial properties and toxicological outcomes becomes impossible. Efforts towards the establishment of consensual minimum levels of analysis of the physicochemical properties of nanomaterials across the entire nanomaterials community have started 2008–2009 [23], however, the full success has not come yet. This paper demonstrates on a practical analysis example, the high degree of standardization of surface analysis workflows with methods completing each other [24]. Figure 1 depicts links of the physicochemical characterization in the framework of risk assessment.

As main surface analytical methods to characterize nanomaterials we chose scanning electron microscopy (SEM), energy dispersive X-ray spectroscopy (EDS), X-ray photoelectron spectroscopy (XPS) and time-of-flight secondary ion mass spectrometry (ToF-SIMS).

The electron microscopy in its various variants, i.e., scanning electron microscopy, transmission electron microscopy (TEM) or combination of the two as scanning transmission electron microscopy (STEM and STEM-in-SEM), constitutes the “working horse” technique for direct visualization of the nanomaterial surface morphology. Further, electron microscopy is well suited for the traceable measurement of the nanoobject size and shape. This valuable capability relies on the calibration of the image magnification, i.e., of the pixel size, by well-known dimensional structures as reference materials [26]. Beyond the great advantage of accessing accurately single nanoparticles, electron microscopy poses challenges mainly related to the representativity of the measurement carried out on a small fraction of nanoobjects (mostly a few hundreds). Particularly for real-world nanomaterials, having mostly a complex morphology, broad particle size distribution and high degree of agglomeration/aggregation, the final result of the particle size distribution can be significantly erroneous [27].

EDS is conventionally a broadly used method applied in combination with a SEM mostly for the quick analysis of the elemental composition of solid samples with a spatial resolution in the range of a micrometer. Technological developments in recent years have enabled the reduction of spatial resolution down to a few nanometers [28,29]. Whilst a quick identification of the main elements in the bulk of the nanoobjects is possible by EDS, the quantitative evaluation of the chemical composition of single nanoobjects is impossible. The relatively quick qualitative information on the presence of elements in the “bulk” of the nanoobjects is very valuable, particularly in combination with the analysis of the morphology of the same nanoobjects by electron microscopy or in combination with other surface chemical analysis techniques such as XPS, ToF-SIMS or Auger electron spectroscopy, with the last technique not included in this study [29,30].

XPS is the most popular surface analysis method and provides information about the chemical composition and the chemical nature of the compounds in the near surface region [31]. Here, the sample is irradiated with X-rays, usually of an energy of 1253.6 (Mg Kα) or 1486.6 eV (Al Kα) and the ejected photo- and Auger electrons are analyzed in an energy spectrometer. The information depth of ca. 10 nm makes this method highly suitable for chemical analysis of nanoparticles.

ToF-SIMS is an even more surface sensitive technique than XPS. In ToF-SIMS, the surface is bombarded by charged species (primary ions) resulting in a collision cascade within the surface near layers. Part of the impetus of the energy transported in the collision cascade is directed towards the surface and atoms and molecules from the uppermost monolayer can overcome the surface binding energy and thus leave the surface. About 1% of the thus sputtered material is charged (secondary ions) and by mass analysis of the ion flux the chemical composition of the uppermost 1–3 nm can be derived. Currently, mainly cluster ion beams (e.g., Bi^3+^) are used for surface excitation and ToF mass analyzers allow the simultaneous detection of elements and intact molecules (<10,000 u) with detection limits in the ppb/fmol range. Semiquantitative information can be gained if the chemical composition (matrix) of the materials to be compared is similar. The key properties are summarized in Table 1. 

As in other areas of science, the “reproducibility crisis” has been discussed in the surface science community, which XPS and ToF-SIMS are part of [32]. Increasing multidisciplinarity, complexity and the high competition between researchers together with limited resources and often wishful thinking were identified as reasons for this crisis. The first two reasons are certainly valid for the risk assessment of nanomaterials. This subject is a paradigm for a highly multidisciplinary and complex research area, which makes it so challenging. As a response of these observations the American Vacuum Society (AVS) begins to develop practical guides for different methods together with renowned scientists. The first guide for XPS has been published recently [33]. It is highly recommendable to consider this guide for work with XPS. In this context, it must be noted, that for the methods presented in this paper there are active standardization bodies, e.g., ISO and CEN (European Committee for Standardization) [34]. Therefore, actual and application-orientated standards are available, and they should be considered in the daily work. The consideration of such guidelines should be a crucial issue for data, which are used for risk assessment.

In this contribution, we present how the quality of physicochemical data of nanomaterials can be ensured in order to use the data for risk assessment. As materials we have chosen Al-coated TiO_2_ nanoparticles, because of the prevalence of titania nanoparticles in consumer and other common products like sunscreens, paints and catalysts. These materials were provided by the Joint Research Centre (JRC) of the European Commission, Ispra (Italy). The focus of the here presented study is to describe the challenges that are accompanied with the surface-analytical characterization of core–shell particles.

## 2. Materials and Methods 

Basically, the physicochemical characterization of nanomaterials with respect to their morphology and chemistry follows the same steps, independent of the type of material: (i) sample preparation, (ii) measurement, (iii) data analysis and (iv) reporting/archiving. The minimum information requirements with respect to the material morphological–chemical characterization of titanium dioxide nanoparticles are described in detail for all the analytical methods selected here: electron microscopy for size and shape, EDS for qualitative bulk elemental composition, XPS and ToF-SIMS for the surface chemistry. Concrete, following two materials have been selected from the JRC repository, see Table 2 [35].

According to the information on the JRC repository [35], both materials selected are rutile having a primary particle size of 21 nm (from XRD measurements) and an Al-coating; one material is hydrophobic (JRCNM62001a) and the other one (JRCNM62002a) hydrophilic. It remains to apply the surface analytical methods selected here and infer the characterization results.

In the present work, a SEM of type Supra 40 (ZEISS, Oberkochen, Germany) with a Schottky field emitter and an InLens secondary electron detector was used at a 5 kV beam acceleration voltage.

The EDS analysis in the present study has been performed with a QUANTAX 400 EDS system (BRUKER, Berlin, Germany), which is equipped with an SDD (Silicon Drift-Detector) of the 10 mm^2^ nominal area. An excitation of 10 keV was applied for the analysis of the titania samples prepared as a thick dry powder layer on an aluminum stub, so that the substrate cannot be coexcited. The analysis areas were selected as large as 5 × 5 µm^2^ on sample agglomerates of about 10 µm size.

XPS measurements were performed at an Axis Ultra DLD (KRATOS, Manchester, UK) with monochromatic Al Kα radiation (E = 1486.6 eV). The electron emission angle was 0° and the source-to-analyzer angle was 60°. The binding energy scale of the instrument was calibrated following a Kratos analytical procedure, which uses ISO 15472 binding energy data [36]. The setting of the instrument was the hybrid lens mode and the slot mode with an analysis area of approximately 300 × 700 µm^2^. Furthermore, charge neutralization with a flood gun was used. All spectra were recorded in the fixed analyzer transmission (FAT) mode. The samples were measured as powders prepared on a special stainless-steel sample holder.

ToF-SIMS analysis was performed on a TOF.SIMS5 instrument (IONTOF, Münster, Germany) in the spectrometry mode, with Bi^3+^ primary ions at an acceleration voltage of 25 keV. The primary ion flux was kept below the static limit (10^12^ ions/cm^2^) and the secondary ions analyzed in both the positive and negative mode. The samples were prepared by fixing the TiO_2_ powder onto a double-sided adhesive substrate (3M removable repositionable tape 665), which was then mounted on the sample holder and introduced to the instrument. Special care was taken to avoid the release of sample material grains into the equipment. This particular adhesive was chosen because it contains significantly less siloxane signals, which result from the coating on the liner.

## 3. Results

### 3.1. Particle Size and Shape

**Figure 2:**
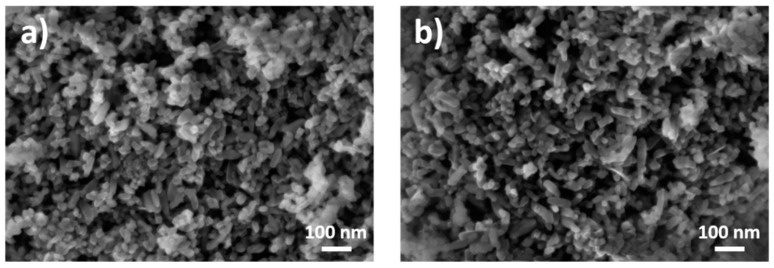
Titanium dioxide nanoparticles imaged by SEM with a secondary electron InLens detector at 5 kV beam voltage: (**a**) JRCNM62001a and (**b**) JRCNM62002a. The samples have been prepared as dry powder on an aluminum sample holder.

Figure 2 displays the SEM micrographs of the two selected titanium dioxide nanoparticulate materials. First, almost all individual nanoparticles are visible. Second, the irregular shape of the particles was also evident, being able to be characterized in a qualitative way as “more or less elongated”. Last, and most challenging is the quantitative evaluation of the particle size distribution. Without any laborious particle size analysis procedure, it is obvious that the mean particle size was well below 100 nm. All the qualitative (but very valuable) observations described above were qualitative. The final descriptive, in fact semiquantitative, result of a good SEM imaging is the following one: both analyzed materials consist of agglomerated/aggregated particles having sizes of about 20–50 nm length and about 20 nm width.

In the vast majority of publications, the most relevant instrumental parameters applied for the measurement of an electron micrograph are specified. These are: beam acceleration voltage, pixel size and pixel number, area of the field-of-view, type of detector, working distance, type of cathode and model of microscope and the type of sample preparation. Other parameters like acquisition time or beam current, sometimes decisive for the accurate visualization of sensitive nanoobjects, are mostly not specified. Beam damage and contamination are often encountered issues at the analysis of nanoparticles. The set of full meta data for SEM micrographs as to be saved in a standardized unique format is in an advanced phase of development at ISO (ISO/FDIS 20171) [37]. Of particular importance for metrological dimensional measurements of nanoparticles by electron microscopy, the traceable calibration of the pixel size should be a requirement in any laboratory. Usually, in accredited laboratories, the calibration state of the electron microscope at well-defined acquisition conditions, i.e., the so-called calibration of image magnification, is performed regularly according to the ISO standard ISO 16700:2016 [38] based on a reference to certified reference materials. The evaluation of the resolution of an SEM is specified in ISO/TS 24597:2011 [39]. Another standardization project addressing more criteria for a complete qualification of a SEM for quantitative measurements is in progress at ISO (ISO/PRF TS 21383) [40].

**Figure 3:**
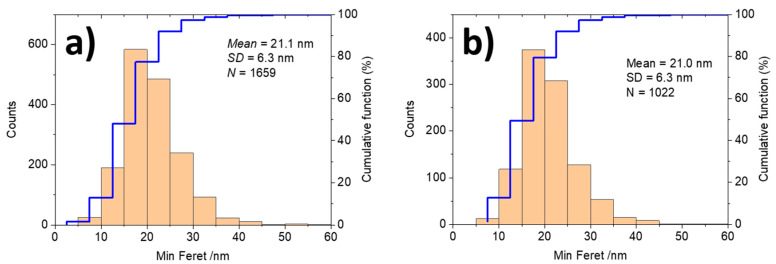
Particle size (minimum Feret diameter) distribution of the titanium dioxide nanoparticles as imaged with SEM in Figure 2: (**a**) JRCNM62001a and (**b**) JRCNM62002a.

A quantitative analysis of the particle size distribution consists of manual identification of the particle boundaries in the electron microscopy micrographs for a large number of particles (mostly at least 500). The result of the particle size distribution measurement for the two samples is shown in Figure 3. For simple cases of spherical and not overlapping nanoparticles automated measurement procedures can be successfully applied. A series of particle size and shape descriptors such as area, equivalent circular diameter, Feret diameter, minimum Feret diameter, roundness, perimeter, aspect ratio, circularity, compactness, perimeter of the convex hull envelope, solidity, ruggedness, etc., can be determined by using various image analysis software packages, some of them being available as part of the SEM or TEM microscope software. The most popular one is ImageJ, a freeware software package developed at the National Institute of Health (USA) [41]. Especially at the tedious manual analysis, the minimum Feret diameter (distance between parallel tangents, corresponds to the “breadth” of the particle) is mostly measured, hence, being also in line with a basic requirement of the EC definition of nanomaterial, which specifies the smallest dimension of particles as defining if a material is nano or not-nano. Meanwhile, there are also software packages available as support for users in categorization of a material as nano or non-nano [42]. The accurate determination of the smallest dimension of particles constitutes a valuable strength of electron microscopy in comparison to other sizing methods when measuring particles of complex shape. The particle sizes evaluated in Figure 3 are expressed as minimum Feret diameter. One should note that also the graphical representation of particle size distribution is standardized, see ISO 9276-1 and -2 [43,44]. Examples of published data represented in this form are given in [27].

One of the most critical points in the accurate measurement of the nanoparticle sizes is the exact knowledge of the threshold for particle segmentation in SEM images. Various thresholding approaches such as Otsu, maximum entropy or IsoData can be applied, e.g., by easy selection from a pop-up menu in ImageJ. A comparative study is presented in [45]. The challenging physical modeling of the electron signals in SEM and the use of reference nanoparticles with known size are key in the evaluation of realistic measurement uncertainties associated with the particle size distribution. In the case of STEM-in-SEM the modeling of the signals of transmitted electrons is more straightforward, see e.g., [46].

The state of the art in the standardized measurement of the nanoparticle size and shape distribution by TEM is given by the very recently published ISO 21363:2020 [47]. Therein not only the exact measurement and analysis protocols (manually and automated) are described, but seven case studies are given in detail in the annexes: discrete spheroidal nanoparticles, size mixture, shape mixture, amorphous aggregates, nanocrystalline aggregates, low aspect ratio nanoparticles and nanoparticles with specific crystal habitats. A similar standard project is still in development as ISO/PRF 19749 [48] for SEM measurement, and includes also representative case studies. An overview with all available ISO standards in use and in development for the measurement of the morphology and chemistry of nanoparticles can be found in [34].

Interlaboratory studies targeted on the accurate measurement of nanoparticle size and shape, but also of other related parameters (including also evaluation of image software) are organized under the prestandardization platform VAMAS (Versailles Project on Advanced Materials and Standards) [49]. The corresponding technical working area TWA 34 “Nanoparticle Populations” has hosted several projects, e.g., [26]. Valuable good proposals can be suggested to VAMAS anytime.

### 3.2. Elemental Composition

**Figure 4:**
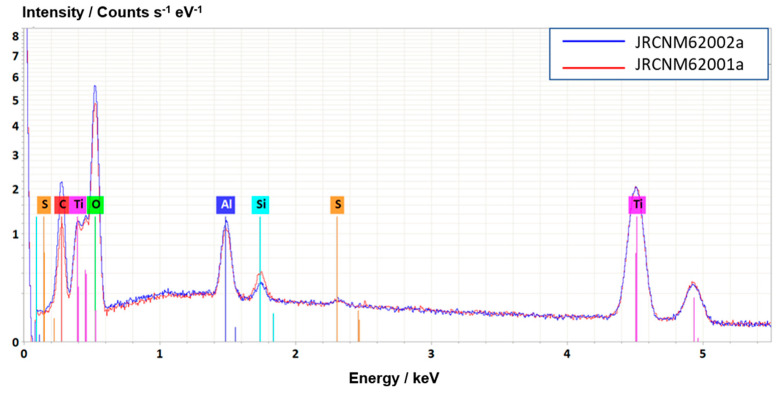
The 10 kV EDS spectra of the titanium dioxide nanoparticles from Figure 3 taken from sample areas of 8 µm × 8 µm: JRCNM62001a (blue) and JRCNM62002a (red). The samples were prepared as dry powder on a silicon wafer. The two spectra have been normalized to the intensity of the Ti Kα peak.

EDS spectra of the two particulate samples prepared as dry powder on a silicon substrate were taken at 10 kV, see Figure 4. The analysis surfaces were selected roughly similarly, so that the direct comparison of the two spectra is allowed. The immediate conclusion is that the materials consist mainly of titanium and oxygen. Further, as expected, aluminum (from the specified coating) and some silicon are also clearly detected. Carbon completes the short tableau of elements quickly and reliably identified as being present in the bulk of both materials. Sulphur is identified in both samples at the detection limit of the method, i.e., about 0.1–0.2 at%. The significant difference between the two EDS spectra lies in the higher intensity of carbon and oxygen in the case of the JRCNM62002a sample, this suggesting the presence of organic compounds—probably responsible for the specified surface hydrophilicity of the material. Note that EDS is not able to distinguish between surface and inner bulk of the nanoparticles, all elements detected come from the “bulk” of the sample. Last interesting observation in the EDS spectra is the somewhat higher intensity of Si in the JRCNM62001a sample, this pointing at Si-containing agents present on the TiO_2_ cores. This observation is confirmed by the XPS and ToF-SIMS analyses, which will be described later. It must be noted that a quantitative analysis of the sample is not possible, because the available quantification algorithm leads to invalid results for nanostructured materials. Therefore, a discussion on the measurement uncertainties is obsolete. As already underlined, even so, the quick qualitative elemental analysis by EDS has gained its dedicated value in the nanoparticle characterization, also due to the direct correlation to the SEM imaging over the same SEM/EDS instrument.

With the knowledge on bulk elemental composition by means of EDS as described above, the next characterization sequence is the “pure” surface chemical analysis by XPS and ToF-SIMS. The corresponding results and correlations to the SEM/EDS results are described in the next two subsections.

Finally, to the elemental EDS analysis, it should be noted that the check of the EDS performance and the measurement and (qualitative and quantitative) analysis of EDS spectra have been standardized already for almost two decades (ISO 15632:2012, ISO 22309:2011) [50,51]. Moreover, the EDS spectra can be saved/exported in files with a unique standardized format (EMSA/MSA, ISO 22029:2012) [52], an option that makes the interchange of EDS data including all relevant raw data, meta data and analysis results working trouble-free in the very large EDS user community.

### 3.3. Surface Chemistry

#### 3.3.1. X-ray Photoelectron Spectroscopy

XPS is a well-standardized method with an active community. An overview about relevant standards has been given recently [53]. First of all, reliable measurements require well-calibrated equipment that can be ensured with the regular procedure of calibration of the binding energy scale according to ISO 15470 [54] and a check of the day-to-day performance (ISO 16129) [55]. It is necessary that the actual performance state of the spectrometer is well documented, preferentially in the metadata accompanying the saved spectrum. Additionally, the type of the spectrometer must be given.

For such sensitive objects like nanoparticles, the preparation procedure for measurements on a substrate in vacuum is crucial for the interpretation of the measurements. Two methods are common for pristine nanoparticles. The first method consists of dispersing and dropping on a silicon wafer. The solvent is evaporated before introducing the sample into vacuum. The other usual method is to measure the pristine (dry) powder. This can be fixed on a double adhesive tape or in a dedicated sample holder with a cavity for the powder. A third method, pressing the powder into a pellet, becomes less popular in the last years, because the sample surface is very likely changed under the high pressure applied. Each method has advantages and disadvantages, which are described in detail elsewhere [56]. In any case, each step of the sample preparation procedure must be specified appropriately. ISO 20579-4 [57] describes the minimum information needed for reporting the handling, preparation and mounting of the samples.

Recording of the XPS spectra is an automatic procedure, at which the settings of the spectrometer have to be adjusted according to the aim of the measurements. An overview about the elements can be obtained with a survey spectrum with a high pass energy (between 50 and 200 eV depending on the spectrometer), whereas for the determination of the valence states high-resolution spectra with a low pass energy (below 30 eV) is advisable. Depending on the lateral homogeneity of the sample, which depends obviously on the sample preparation, measurements with a large spot with a size of hundreds of micrometers or with a selected area down to 10 µm spot size should be chosen. The analysis area influences the setting of the electrooptical lenses, which are standard for modern spectrometers. Furthermore, for insulating samples charge compensation is used for the spectrometer recording. Of course, the acquisition time or the number of steps, the number of sweeps and the step size, and the dwell time (time per step) are also important. All this information is necessary for the reproducibility of the measurements. Additionally, a reliable data reduction and interpretation are not possible without this exact knowledge of the measurement conditions. For example, it makes no sense to determine a binding energy with a precision of a tenth of an eV for measurements with a step size of 1 eV. In the VAMAS format the most of this information is given. The base pressure of the UHV can give some information about adsorbates from the residual gas.

**Figure 5:**
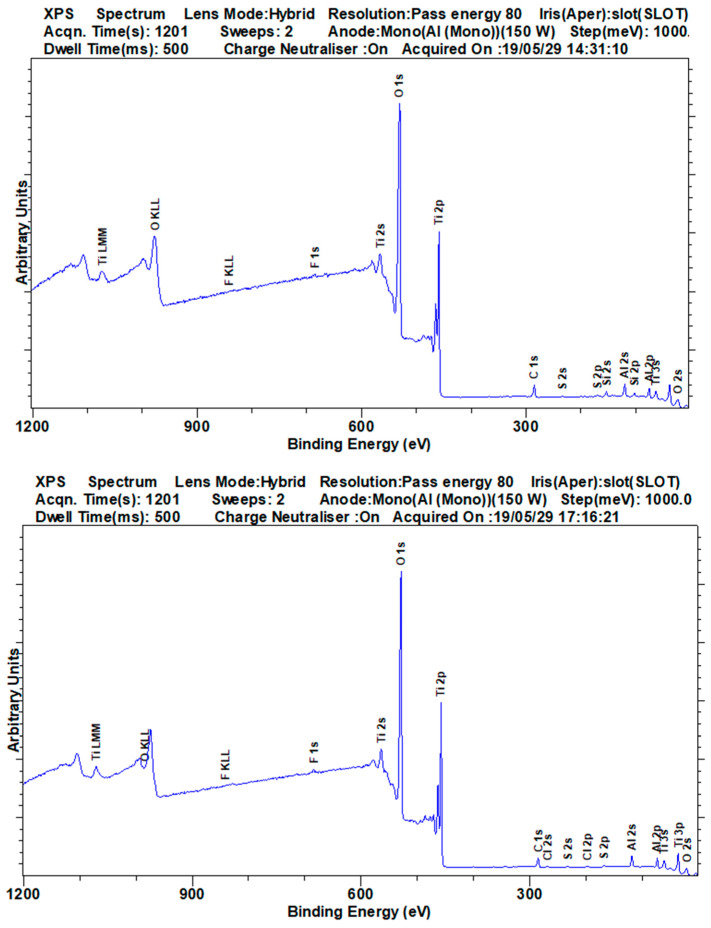
Survey XPS spectra of JRCNM62001a and JRCNM62002a titania nanoparticles with corresponding Versailles Project on Advanced Materials and Standards (VAMAS) information on top.

Figure 5 shows a survey spectrum with the corresponding information in VAMAS format on the top. Additionally, all relevant peaks are annotated. Whereas the recording of the spectra is an automatic process and most of the necessary information is given in the VAMAS file format [58,59] without any regard of the user, the annotation of the peaks needs a critical evaluation by the analyst. Before this step, it should be checked if charge correction is necessary. The spectra are referenced to an internal standard. Usually, the main C1s peak is used with a binding energy between 284.5 and 285.0 eV. If this C1s peak cannot be used due to different reasons, another suitable peak of a chemical stable component can be chosen, e.g., the major peak of the cation of the oxidic substrate. The information about the internal charging, which is crucial to reproduce the assignment of the photoelectron peaks and, even more, the correlation of the different states with valence states is described in ISO 19318 [60].

Most software packages offer automatic peak finding options, but especially overlapping peaks and signals close to the detection limit can lead to wrong peak identification. Two principles shall be taken into account: (i) every peak shall be annotated and (ii) the main peaks of an element and each peak of the major components shall be marked in the spectra. Criteria for the certain detection of a signal are well established in the literature [61,62], e.g., the signal should be three times more intense than the noise. The detection limits of XPS are generally given with values between 1 and 0.01 at%, depending on the element and the matrix. Unclear annotations due to peak overlapping or a worse signal-to-noise ratio shall be indicated clearly. In the survey spectrum presented in Figure 5, O, Ti, C, Si and Al can be clearly identified as components of the sample. Complementary investigation with methods with better detection limits like ToF-SIMS can be performed to eliminate any doubts.

**Figure 6:**
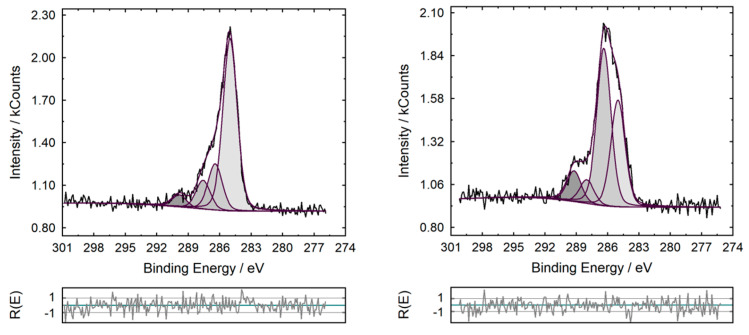
High-resolution C1s spectra of JRCNM62001a (**left**) and JRCNM62002a (**right**), with the corresponding fit parameters.

Valence states can be obtained from the high-resolution spectra. First, a suitable background is subtracted. Different approximations for the background can be used, e.g., linear, Shirley or Tougaard (ISO TR 18392) [63,64]. At least, the chosen background function should be reported and the spectra with the background shown, see the high-resolution spectra in Figure 6. The peak fits with the single components, the sum curve and the difference between the measured and sum curve (residual) are crucial to check the quality and the reliability of the fit. The peaks are usually fitted with mixed Gaussian-Lorentzian curves, whereby products, sums or convolution functions can be used [65]. This must be considered in the reports’ text. In a table the chosen function with the electron binding energies, the Gaussian and Lorentzian amount, the full width at half maximum (FWHM) and the intensities of the identified peaks should be presented. Valence states should be correlated with suitable binding energies on the basis of suitable reference materials or reference measurements. For this correlation the typical uncertainty of ±0.2 eV for the binding energy has to be considered. The high-resolution C1s spectra presented in Figure 6 show that JRCNM62001a and JRCNM62002a consist of different organic species: at JRCNM62001a hydrocarbons or siloxane species are the major part, whereas at JRCNM62002a oxygen-containing carbon species are present with a higher intensity. The presence of siloxanes can be confirmed with the high-resolution Si 2p spectra with a peak maximum at 102.6 eV. This finding corresponds to the hydrophobic character of JRCNM62001a and the hydrophilic properties of JRCNM62002a, which are affected by the outermost organic shell. For the fitting sum, Lorentzian–Gaussian functions were used, which are a good approximation for the real convolution [66]. The fitting parameters are given in Table 3 and Table 4.

The most difficult part of the data reduction in terms of reliability is the quantification of identified peaks into chemical composition. Usually, net peak areas are used, which are obtained after background subtraction. It can be decided if either the survey or the high-resolution spectra are used, but the results cannot be mixed. For the quantification, three approaches can be used: (i) experimentally determined relative sensitivity factors [67,68], (ii) theoretically derived sensitivity factors [69] and (iii) specific reference samples. Reference samples are often not available; thus, this latter approach is often not possible, therefore, the other two approaches are discussed further. Experimentally derived sensitivity factors are provided by some manufacturers for their instruments and can be easily used. In contrast, theoretically derived sensitivity factors are published, verified and can be transferred between different instruments and modes. In this approach, the peaks are normalized with the element-specific Scofield relative sensitivity factors, the mean free path length of the photoelectrons and the spectrometer-dependent transmission function [70]. In Table 5, results obtained with the two different approaches are compared. For significant peaks, a relative uncertainty for a confidence interval of 95% below 15% was determined, for traces below 1 at% a quantification is not reliable. These are values that are discussed as uncertainties in the literature, thus, it can be concluded that both approaches led to similar results. Despite this finding, it is necessary to specify the method, which was used for the quantification.

Both models have one major disadvantage in common. They assume homogeneous material composition in the analysis volume, which is not fulfilled for nanoobjects. They do not consider inhomogeneities like size and morphology effects, core–shell structures, etc. [71]. Therefore, it is unclear if the carbon species with a similar amount exist as a homogeneous layer and as three-dimensional islands on the shell of the nanoparticles. A deeper look at the background can answer this question [72].

In recent years, great efforts were undertaken to develop more suitable methods for the accurate chemical analysis of nanoparticles [73,74]. Especially, for the analysis of organic components and core–shell structures, these approaches deliver access to new promising opportunities [75,76,77]. For the nanoparticles described in this study, a rough estimation was performed for determining the thickness of the Al shell with simulation of electron spectra for surface analysis (SESSA). Spheres with a diameter of 20 nm were assumed. A thickness below 1 nm was found for the alumina shell. This observation of a very thin layer was confirmed by the ToF-SIMS results detecting Ti- and Al-containing ions. Further details about the simulation are given in the Appendix A (Table A1, Table A2 and Table A3). Of course, a detailed documentation of the assumptions and the values taken in such determination or simulation is necessary, e.g., the used effective attenuation length, densities, Scofield factors, etc. In every case, we recommend showing the (relative) intensities of the peaks corrected by the transmission function. If such calculations or simulations of the thicknesses of shells were performed, it allows one to trace back the determination of the thickness; if not, the experimental data can be used by other groups, which are able to perform such calculations. Thus, the producers of the experimental data benefit from citations of their study.

#### 3.3.2. Time-of-Flight Secondary Ion Mass Spectrometry (ToF-SIMS)

**Figure 7:**
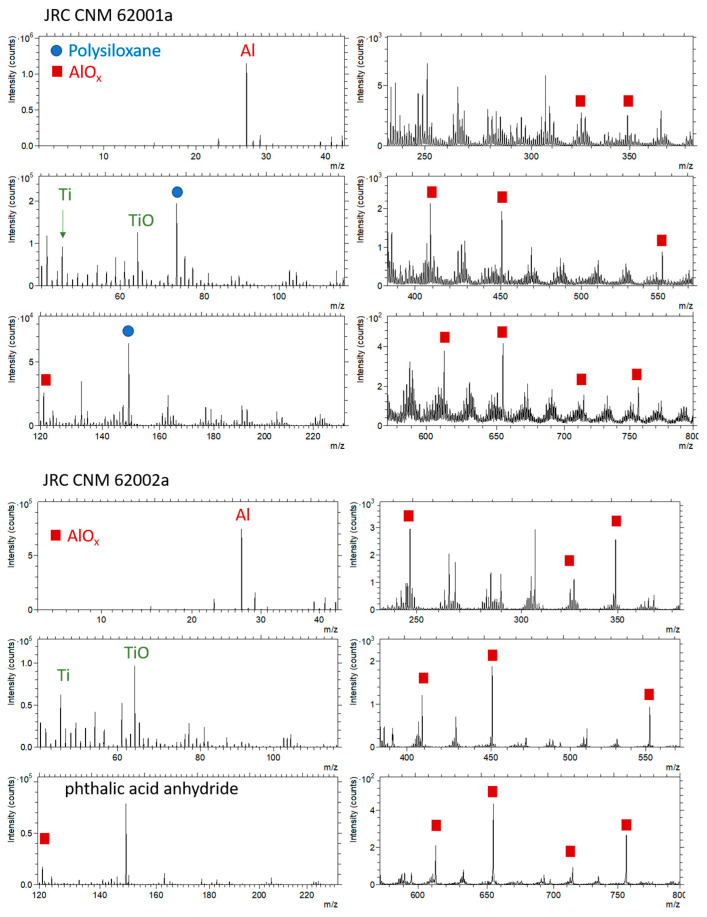
ToF-SIMS spectra, positively charged secondary ions (**top**: JRCNM 62001a, **bottom**, JRCNM 62002a); field of view: 325 × 325 µm^2^.

**Figure 8:**
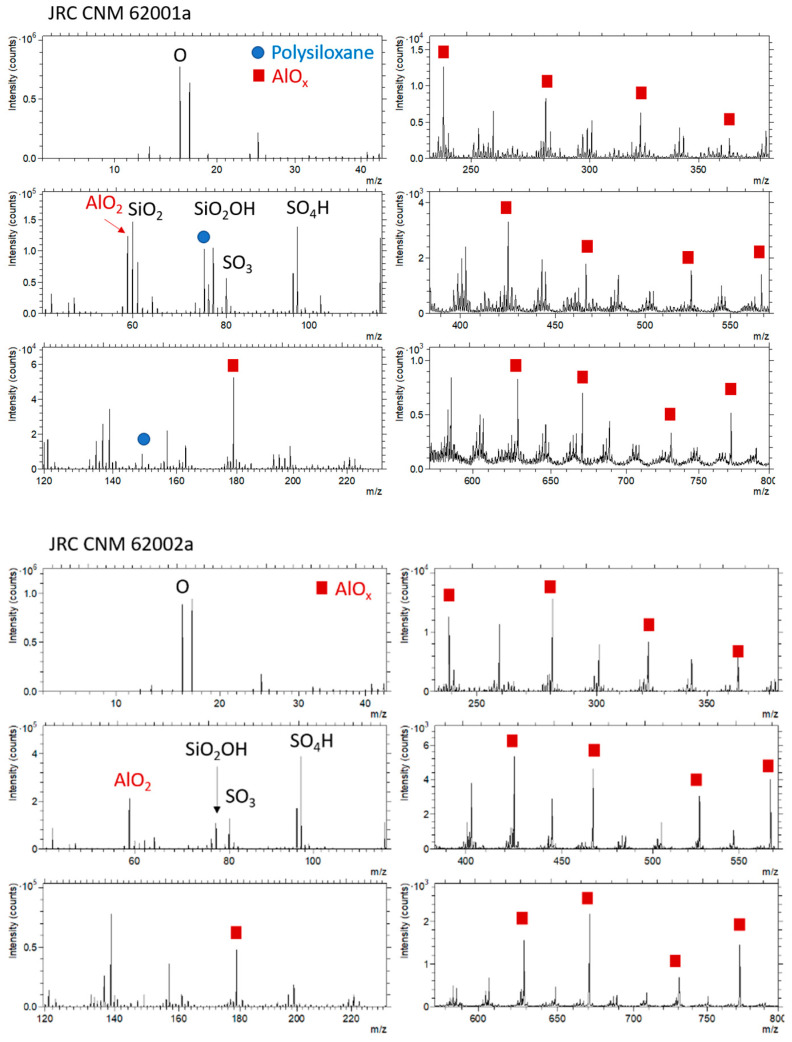
ToF-SIMS spectra, negatively charged secondary ions (**top**: JRCNM 62001a, **bottom**, JRCNM 62002a); field of view: 325 × 325 µm^2^.

ToF-SIMS is the most surface sensitive technique amongst the techniques considered here. The information mainly is originating from the uppermost 1–3 atomic or organic layers (“1–3 monolayers”). Figure 7 shows the spectra of the positively charged secondary ions and Figure 8 the spectra of the negatively charged secondary ions. The assignment of the peaks is focused on particular peaks in the context of the analysis of the coating and of the Al_2_O_3_ shell on the TiO_2_ nanoparticles. Furthermore, peaks showing significant differences between both samples are labeled.

For both particles, Al and Ti along with their oxides can be detected. The presence of Ti-containing ions suggests that either the covering AlOx layer was thinner than the SIMS information depth or the AlOx coating was not completely closed. Table 6 shows that there was no significant difference in the Ti+/Al+ ratio for the 2 particles, which is in accordance with the assumption of similar primary particles having a similar AlOx coating. Differences exist with respect to coverage with organic materials and further inorganic molecules.

Whereas for the particles JRCNM 62001a polysiloxanes, possibly as mixtures with silane-like material, can be detected, for the particles JRCNM 62002a no evidence for the presence of glycerol could be found. Not only are significant glycerol signals missing in the positive polarity, but also fatty acid residues can hardly be detected in the negative polarity. Nevertheless, O-containing hydrocarbons are found with higher intensities for JRCNM 62002a (Table 6). Instead of glycerol, sulfates and phthalic acid anhydride are detected with significant intensities (Table 6). Whether the phthalic acid compound is a contaminant (e.g., adsorption from ambient air) or deliberately administered to the surface needs to be discussed with the producer. The phthalic acid may also be the cause for the higher intensities of the O-containing hydrocarbons.

Differences also exist with respect to the hydrocarbon intensities. Whereas the intensities of aromatic hydrocarbons are higher for the particles JRCNM 62001a, the intensities of aliphatic hydrocarbons are higher for the particles JRCNM 62002a. Given the field-of-view of 325 × 325 µm^2^, the data represent an average over many particles. Data from three repetitive measurements showed no significant differences between the analysis positions.

ToF-SIMS gives detailed information on both the inorganic and organic composition of the outer monolayers of the nanomaterials. Additionally, semiquantitative information could be gained concerning the differences in chemical composition at the outermost surface of the nanoparticles.

## 4. Conclusions

**Figure 9:**
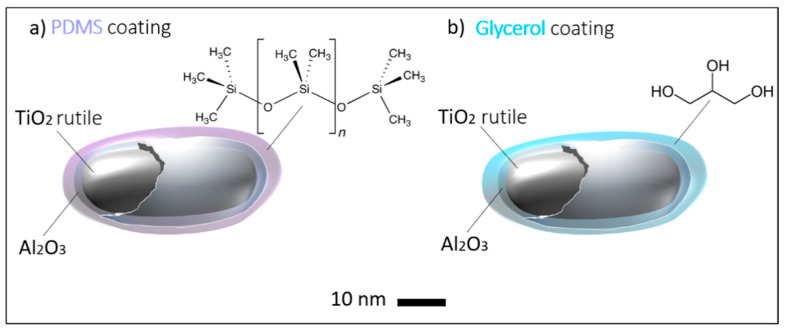
Schematic representation of core–shell Al-coated titania nanoparticles with different types of functionalization layers: (**a**) PDMS coating and (**b**) glycerol coating. The shape of the particles can vary, however, these are not a round shape. For the approximate size estimation, a nanometer scale bar is displayed.

A deep insight into the physicochemical nature of core-shell nanoparticles was obtained by combining the four surface analytical methods SEM, EDS, XPS and ToF-SIMS, see the schematic illustration in Figure 9. Whereas SEM allows the determination of the size and shape of the particles, information about the chemical composition was obtained with EDS (bulk) and XPS (surface). A simple quantification assuming homogeneous chemical composition is not sufficient to get reliable results about the quantitative composition of the particles by both methods EDS and XPS. However, with XPS reliable simulation of the quantitative composition is possible. Due to different information depths and detection limits, XPS and ToF-SIMS are complementary methods, which allow a different view on the surface chemistry of the particles. The XPS results suggest an Al_2_O_3_ coating of less than 1 nm. The appearance of Ti containing ions in the SIMS spectra confirms this result. Based on our observations it is not possible to exclude some inhomogeneities of the shell like an imperfect coating. It is obvious that for the specification of the surface chemistry the titania core should be specified additional to the organic coating and the Al_2_O_3_ shell as an important endpoint for the risk assessment.

Only with such a complementary experimental approach a comprehensive physico-chemical analysis of the nanoparticles becomes possible. For a reliable analysis the consideration of already available standards and guidelines reflecting the state-of-the-art and a detailed documentation of all steps in the analysis is required, so that based on a minimum of mandatory information the results of the analysis can be reproduced at any time in any laboratory.

The current type of high-quality data obtained through standardized and FAIR procedures provide a robust basis for advanced next-generation, in silico-driven risk assessment enabling grouping and read across. As discussed above, key challenges with these approaches within nanotoxicology have been, among other things, the lack of standardized approaches for testing and assessment. The physicochemical data presented herein has the potential to, together with for example high-throughput toxicological data providing indications of perturbed toxicity pathways, raise the regulatory approaches for grouping and read across to new levels of efficiency and accuracy [78]. Such a scenario has been shown previously within the field of chemical toxicity, whereby integration of information on chemical structure and biological activity significantly increases the accuracy of read across-based toxicity prediction, and additionally provides insight into the mechanism of action [79]. Finally, these approaches significantly contribute to reduced needs for extensive testing in animals for the purpose of risk assessment [80].

## Figures and Tables

**Table 1:** Key properties of the methods.

Method	SEM	EDS	XPS	ToF-SIMS
Physico-chemical properties	Size, shape	Elemental composition	Composition, surface chemistry	Surface chemistry
Lateral resolution	1–2 nm	<1 µm	>1 µm	50 nm
Information depth	a few nm	<1 µm	10 nm	1–3 nm
Quantification	Length of projection dimensions	No quantification for single nanoparticles	With suitable models	challenging
Detection limit	a few nm	0.1 at%	0.01–1 at%	ppb

**Table 2:** Main physicochemical parameters of the titanium dioxide material according to the Joint Research Centre (JRC) repository.

JRC ID	Type of Material	Primary Crystallite Size (nm) ^1^	Other Information
JRCNM62001a	titanium dioxide	21	rutile, thermal, hydrophobic, Al-coated
JRCNM62002a	titanium dioxide	21	rutile, thermal, hydrophilic, Al-coated

^1^ from XRD.

**Table 3:** Fit parameters of the C1s of JRCNM62001a.

Peak Name C1s	Peak Height/Counts	Lorentzian	Position/eV	FWHM/eV	Abs. Area/Counts*eV	Rel. Area/%
C-C, C-H	1217.3	0.2	285.0	1.6	2244	66.3
C-O	327.4	0.2	286.4	1.6	604	17.9
C=O	203.9	0.2	287.6	1.6	376	11.1
O-C=O	86.0	0.2	289.9	1.6	159	4.7

**Table 4:** Fit parameters of the C1s of JRCNM62002a.

Peak Name C1s	Peak Height/Counts	Lorentzian	Position/eV	FWHM/eV	Abs. Area/Counts*eV	Rel. Area/%
C-C, C-H	647.8	0.2	285.0	1.6	1194	33.5
C-O	955.8	0.2	286.4	1.6	1763	49.4
C=O	143.7	0.2	288.0	1.6	265	7.4
O-C=O	187.3	0.2	289.2	1.6	645	9.7

**Table:** 

JRCNM62001a	O 1s	Ti 2p	Si 2p	C 1s	F 1s	Al 2p
Exp.	64.2	14.8	2.2	8.9	0.8	9.2
Sco	62.9	16.1	2.5	7.2	0.5	10.8
U (*k* = 2)	0.8	0.8	0.2	1.0	0.2	0.9
u	1.3	5.2	8.5	12.5	30.8	9

**Table:** 

JRCNM62002a	O 1s	Ti 2p	C 1s	F 1s	Al 2p	Cl 2p	S 2p
Exp.	64.2	16.3	7.8	0.6	9.7	0.4	0.6
Sco	61.8	17.3	7.5	0.4	11.8	0.4	0.8
U (*k* = 2)	1.4	0.6	0.2	0.2	1.2	0	0.1
u	2.2	3.6	2.6	40.0	11.2	0	20.0

**Table 6:** Intensity ratio between the particles JRCCNM 62001a (1) and JRCNM 62002a (2); data normalized to Al^+^ and AlO_2_^−^, respectively.

Secondary Ions	Ratio 1/2
Ti^+^	1.09
C_x_H_y_, aliph.	0.70
C_x_H_y_, arom.	1.47
siloxanes	135.00
phthalate	0.58
C_x_H_y_O_z_	0.7
SiO_2_^−^	18.08
SO_3_^−^	0.68

## Data Availability

The data presented in this study are available on request from the corresponding author. The data are not publicly available due to privacy.

## Appendix A

Input parameters for SESSA (simulation of electron spectra for surface analysis version 2.1.1) by the National Institute of Standards and Technology (NIST), Standard Reference Database Program SRD 100. The manual is available free of charge from: https://doi.org/10.6028/NIST.NSRDS.100-2017.

**Table A1:** Areas of the peaks corrected with the intensity–energy response function (IERF) of the spectrometer. These values were used for the calculation with simulation of electron spectra for surface analysis (SESSA). The estimated uncertainty is up to 10% with a confidence level of 95%.

Peak Name	Ekin/eV	Area/cps*eV	IERF	Corr. Area/cps*eV
**JRCNM62001a**				
**Ti 2p**	1027.2	160574.6	1082.7	148.3
**Al 2p**	1411.2	6362.6	745.2	8.5
**JRCNM62002a**				
**Ti 2p**	1028.9	191327.7	1081.3	176.9
**Al 2p**	1412.9	7710.9	743.9	10.4

**Table A2:** Input parameters for SESSA and mean free paths (IMFP: inelastic mean free path; EMFP: elastic mean free path).

Input-Parameters: Single-nanoparticle Model.			
**Morphology: Layered Spheres; z-height:100.0 Å; x-period: 250 Å; y-period: 250 Å**			
	**Density/g/cm^3^**	**Thickness/Å**	**Energy Band Gap/eV**
Al_2_O_3_	3.97	10.0	10.0
TiO_2_	4.17	190.0	5.0
	**Peak Position Ekin/eV**	**Anisotropy β**	**Cross Section/Å^2^**
Al 2p 1/2	1413.7	0.95	2.463 × 10^−5^
Al 2p 3/2	1414.1	0.95	4.842 × 10^−5^
Ti 2p 1/2	1026.4	1.40	3.658 × 10^−4^
Ti 2p 3/2	1032.8	1.40	7.099 × 10^−4^
**IMFP/Å**	**in Al_2_O_3_**	**in TiO_2_**	
Al 2p 1/2	33.92		
Al 2p 3/2	33.93		
Ti 2p 1/2	26.52	22.27	
Ti 2p 3/2	26.65	22.38	
**EMFP/Å**	**in Al_2_O_3_**	**in TiO_2_**	
Al 2p 1/2	13.31		
Al 2p 3/2	13.31		
Ti 2p 1/2	10.44	10.17	
Ti 2p 3/2	10.49	10.21	
**Geometry**			
	**phi**	**theta**	**rot**
Sample surface normal	0°	0°	0°
Analyzer axis	0°	0°	
Source axis	0°	60°	
Polarization vector	0°	0°	
aperture	0°–360°	0°–15°	

Calculations:

- Convergence factor: 1 × 10^−5^; straight line approximation
- Number of collisions: auto (50); number of trajectories: auto (100211)

**Table A3:** Calculated peak intensities.

Ti 2p 1/2	Ti 2p 3/2	Al 2p 1/2	Al 2p 3/2
4.479 × 10^−7^	8.641 × 10^−7^	2.619 × 10^−8^	5.140 × 10^−8^
